# A novel risk model of three SUMOylation genes based on RNA expression for potential prognosis and treatment sensitivity prediction in kidney cancer

**DOI:** 10.3389/fphar.2023.1038457

**Published:** 2023-05-02

**Authors:** Song-Chao Li, Li-Jie Yan, Xu-Liang Wei, Zhan-Kui Jia, Jin-Jian Yang, Xiang-Hui Ning

**Affiliations:** ^1^ Department of Urology, The First Affiliated Hospital of Zhengzhou University, Zhengzhou, China; ^2^ Institute of Pharmaceutical Science, Zhengzhou University, Zhengzhou, China

**Keywords:** kidney cancer, prognosis, sumoylation, targeted therapy, immune threapy

## Abstract

**Introduction:** Kidney cancer is one of the most common and lethal urological malignancies. Discovering a biomarker that can predict prognosis and potential drug treatment sensitivity is necessary for managing patients with kidney cancer. SUMOylation is a type of posttranslational modification that could impact many tumor-related pathways through the mediation of SUMOylation substrates. In addition, enzymes that participate in the process of SUMOylation can also influence tumorigenesis and development.

**Methods:** We analyzed the clinical and molecular data which were obtanied from three databases, The Cancer Genome Atlas (TCGA), the National Cancer Institute’s Clinical Proteomic Tumor Analysis Consortium (CPTAC), and ArrayExpress.

**Results:** Through analysis of differentially expressed RNA based on the total TCGA-KIRC cohort, it was found that 29 SUMOylation genes were abnormally expressed, of which 17 genes were upregulated and 12 genes were downregulated in kidney cancer tissues. A SUMOylation risk model was built based on the discovery TCGA cohort and then validated successfully in the validation TCGA cohort, total TCGA cohort, CPTAC cohort, and E-TMAB-1980 cohort. Furthermore, the SUMOylation risk score was analyzed as an independent risk factor in all five cohorts, and a nomogram was constructed. Tumor tissues in different SUMOylation risk groups showed different immune statuses and varying sensitivity to the targeted drug treatment.

**Discussion:** In conclusion, we examined the RNA expression status of SUMOylation genes in kidney cancer tissues and developed and validated a prognostic model for predicting kidney cancer outcomes using three databases and five cohorts. Furthermore, the SUMOylation model can serve as a biomarker for selecting appropriate therapeutic drugs for kidney cancer patients based on their RNA expression.

## Introduction

Kidney cancer is one of the most common and lethal urological malignancies. Clear cell renal cell carcinoma (ccRCC) composes approximately 70%–85% of all kidney cancers and arises from the epithelium of the proximal tube of the kidney (Li et al., 2019a). Patients with ccRCC have a poor outcome, although targeted therapy and immune checkpoint blockade (ICB) agents have been recommended as first-line treatment for metastatic ccRCC (Albiges et al., 2019). More accurate prognosis prediction and personalized treatment plans are essential for the full-course management of patients with ccRCC. Today, many risk models that consist of clinical and molecular features have been proposed to predict the outcome in patients with kidney cancer (Wei et al., 2019; Ning et al., 2021; Li et al., 2022).

Posttranslational modification (PTM) is a process to modify polypeptide chains through chemical modifications, which makes protein functions more diverse. About 400 types of PTMs have been reported. SUMOylation is carried out through small ubiquitin-related modifier (SUMO) proteins and enzymes that connect SUMO proteins to a lysine residue in the target protein (Ramazi and Zahiri, 2021). SUMOylation has multiple physiological functions, such as chromatin remodeling, stemness, cell identity, and protein stability. Abnormal SUMOylation has been proven to be associated with cancer occurrence, neurodegeneration, and infection (Celen and Sahin, 2020). In cancers, SUMOylation of promyelocytic leukemia/retinoic acid receptor alpha (PML/RARA) is important for cellular transformation and essential for the pathogenesis of acute promyelocytic leukemia. Androgen receptor SUMOylation is associated with tumorigenesis of prostate cancer, while SUMOylation of proto-oncogene MYC occurs in B cell lymphoma (Sahin et al., 2022). SUMOylation of tumor suppressor gene p53 might have two opposite functions in cancer, cancer promotion, and cancer suppression (Bettermann et al., 2012). Some drugs that target SUMOylation have been discovered. Ginkgolic acid could reduce cellular SUMOylation and inhibit the invasion capacity of lung, colon, and liver cancers (Baek et al., 2017; Qiao et al., 2017; Li et al., 2019b; Sahin et al., 2022).

In kidney cancer, transcription factors, hypoxia-inducible factor 1A (HIF1A) and hypoxia-inducible factor 2A (HIF2A) are important for tumorigenesis and development of ccRCC through activating transcription of genes, such as vascular endothelial growth factor, which could impact the angiogenesis signaling pathway (Schodel et al., 2016). Previous studies have shown that SUMOylation could also take part in the carcinogenesis of kidney cancer. SUMOylation of HIF1A, E3 ligase hypoxia-associated factor could bind to HIF2A and enhance its transcriptional activity, which then promotes the metastasis of ccRCC cells (Koh et al., 2015). In addition, mutation of microphthalmia-associated transcription factor (MITF) could impair the SUMOylation of MITF, and then improve the transcriptional activity of HIF1A through binding to its promoter (Bertolotto et al., 2011). A study has also shown that SUMOylation-related genes could help predict the outcome of patients with bladder cancer (Guo et al., 2022). However, the role of SUMOylation-related genes have not been well discussed in kidney cancer.

In this study, we screened the differentially expressed SUMOylation genes at the RNA level between kidney cancer and normal kidney tissues. A SUMOylation risk model was constructed to predict prognosis and guide the drug selection for the treatment of patients with kidney cancer. We then found the different immune statuses between various SUMOylation risk groups and analyzed protein expression further. This study not only built a robust prognosis model but also screened the significant SUMOylation-related genes, which may be essential for the development of kidney cancer.

## Methods

### Data acquiring

Three databases were used in this study: The Cancer Genome Atlas (TCGA), the National Cancer Institute’s Clinical Proteomic Tumor Analysis Consortium (CPTAC), and ArrayExpress. Specifically, the Kidney Renal Clear Cell Carcinoma (KIRC) cohort in the TCGA database, PDC000127 cohort in the CPTAC database, and E-MTAB-1980 cohort in the ArrayExpress database were enrolled in our study (Sato et al., 2013; Clark et al., 2019). Clinical and RNA expression data of these three cohorts, and protein expression data of the PDC000127 cohort and Chinese Fudan cohort were downloaded and processed (Clark et al., 2019; Qu et al., 2022). Data of patients who had complete clinical data in the three cohorts were used for prognosis analysis. Detailed patient lists and clinical features are shown in the supplementary file and summarized in Supplementary Table S1. The SUMOylation genes were obtained from the molecular signature database (Supplementary Material).

### Analysis of differential SUMOylation gene expression

RNA-seq data from 72 normal kidney tissues and 539 tumor tissues in the TCGA-KIRC cohort were used to screen the differential SUMOylation gene expression. The “limma” package was used, and genes with a fold change>1 and false discovery rate <0.05 were considered differentially expressed (Ritchie et al., 2015). The intersection of differential SUMOylation genes, and the TCGA-KIRC, CPTAC, and E-MTAB-1980 gene lists were used for further clinical prognosis analysis.

### SUMOylation-related gene risk model construction

In total, 513 TCGA-KIRC patients, 89 CPTAC patients, and 99 E-MTAB patients were diagnosed with ccRCC by pathology, and with complete clinical data (age, sex, clinical stage, tumor stage, metastasis stage, node stage, grade, and survival time more than 1 month), were analyzed for building a prognosis risk model. First, the 513 TCGA-KIRC patients were randomly separated into a discovery TCGA cohort (256 patients) and a validation TCGA cohort (257 patients) in a 1:1 ratio, using the set.seed function of Base R, and the seed was set as 4443. The discovery TCGA cohort was used for constructing a risk model, and the differential SUMOylation genes were analyzed by univariate cox regression to find the prognosis-related genes. These genes were analyzed with Lasso regression analysis, and the selected genes were tested in multivariate cox regression analysis using the Akaike information criterion method. The coefficient and expression value of these genes were employed to compute a risk score and construct a risk model, which was named the SUMOylation risk model in this study.

The risk score of each patient in the five cohorts (discovery TCGA cohort, validation TCGA cohort, total TCGA cohort, CPTAC cohort, and E-MTAB-1980 cohort) was calculated. Patients in each cohort were allocated into high- and low-risk groups according to the median SUMOylation risk score as the cut-off value. Outcomes of the different groups of patients were evaluated by log-rank analysis and presented by Kaplan-Meier curve, and the receiver operating characteristic curve was conducted. The propensity-matched analysis (PSM), subgroup analysis, and interaction test was conducted to further test and validate the role of the risk group in outcome predicting. The PSM test was conducted using the “MatchIt” package and the caliper was set as 0.03. The role of SUMOylation risk score in prognosis prediction was investigated by univariate and multivariate cox regression sequentially. In addition, a nomogram was built, and then multi-ROC and calibration analyses were conducted to test the nomogram.

### The correlation between the SUMOylation risk group and molecular features

Gene Set Variation Analysis (GSVA) analysis which was conducted by “c2. cp.kegg.v6.2. symbols.gmt” and “GSVA” package was used to clarify the molecular features in the tumor tissues of different risk groups. The infiltration immune cell proportion in tumor tissues of TCGA-KIRC patients was computed and assessed using CIBERSORT (https://cibersort.stanford.edu/) (Newman et al., 2015).

In addition, the immune subtype was raised according to the 160 immune expression signatures, and the all-type tumors in the TCGA database were clustered into six subtypes, C1 (wound healing), C2 (IFN-g dominant), C3 (inflammatory), C4 (lymphocyte depleted), C5 (immunologically quiet), and C6 (TGF-b dominant) (Thorsson et al., 2018). Subtype data of the TCGA-KIRC tumors were taken out.

A previous study proposed and computed the TIDE score of samples across tumor types in the TCGA database based on the expression of specific genes. The results are published at https://tide.dfci.harvard.edu/, and related information of TCGA-KIRC patients was extracted from there (Jiang et al., 2018).

Drug treatment sensitivity predicting information was packaged in the “pRRophetic” package (Yang et al., 2013; Geeleher et al., 2014). The targeted agent’s treatment sensitivity of TCGA-KIRC patients was calculated using the package and represented half maximal inhibitory concentration (IC50).

The SUMOylation risk model genes encoding protein expression data of CPTAC and Chinese Fudan cohort were obtained from the published data (Clark et al., 2019; Qu et al., 2022). The IHC data and images of renal cancer and kidney tissues were obtained and analyzed using the HPAnalyze package (Tran et al., 2019).

### Immunohistochemistry (IHC) detection and IHC score

The tissue microarray (TMA) containing 30 kidney cancer tissues and 30 adjacent normal kidney tissues was purchased from SHANGHAI OUTDO BIOTECH CO., LTD (Lot: HKid-CRCC060PG-01). Primary antibodies targeting CDCA8 (PROTEINTECH: 12465-1-AP), CDH1 (PROTEINTECH: 20874-1-AP), and PPARA (PROTEINTECH: 66826-1-Ig) were used to detect the corresponding protein expression via IHC. The IHC procedure involved deparaffinization and rehydration of the TMA, followed by heating the slides in Tris-EDTA (PH 8.0) for antigen retrieval. Subsequently, the slides were incubated in 0.3% H_2_O_2_ for 30 min and blocked in 10% goat serum to prevent non-specific binding. The slides were then incubated with antibodies against CDCA8 at a 1:100 dilution, CDH1 at a 1:600 dilution, and PPARA at a 1:100 dilution overnight at 4°C. Finally, the slides were incubated with the secondary antibody and visualized using DAB chromogen.

We used ImageJ software to analyze the staining intensity of IHC. We measured the staining intensity for each tissue section and divided the samples into quartiles. Based on these quartiles, we classified the samples into three groups: low expression (staining intensity below the 25th percentile), moderate expression (staining intensity between the 50th and 75th percentiles), and high expression (staining intensity above the 75th percentile).

### Statistical analysis

Continuous variables were analyzed by the t-test, while categorical variables were examined using the chi-square test. All statistical analyses were conducted using R software, and a two-sided *p*-value of < 0.05 was considered statistically significant.

## Results

### Differential SUMOylation genes in TCGA-KIRC dataset

Among these SUMOylation genes, 29 genes were abnormally expressed in kidney cancer tissues. Of these, 17 genes (CDKN2A, H4C5, AURKB, BIRC5, H4C11, TOP2A, cell division cycle-related protein 8 [CDCA8], H4C12, NR5A2, BLM, NFKB2, PML, H4C8, H4C9, RARA, NR1H3, and H4-16) were upregulated and 12 genes (CHD3, peroxisome proliferator-activated receptor alpha [PPARA], NR4A2, CDH1, VDR, PPARGC1A, THRB, NR3C2, TFAP2A, TFAP2C, NOS1, and TFAP2B) were downregulated than in the adjacent normal kidney tissues (Supplementary Material; Supplementary Figure S1).

### SUMOylation gene risk model for predicting the survival of patients with kidney cancer

**FIGURE 1:**
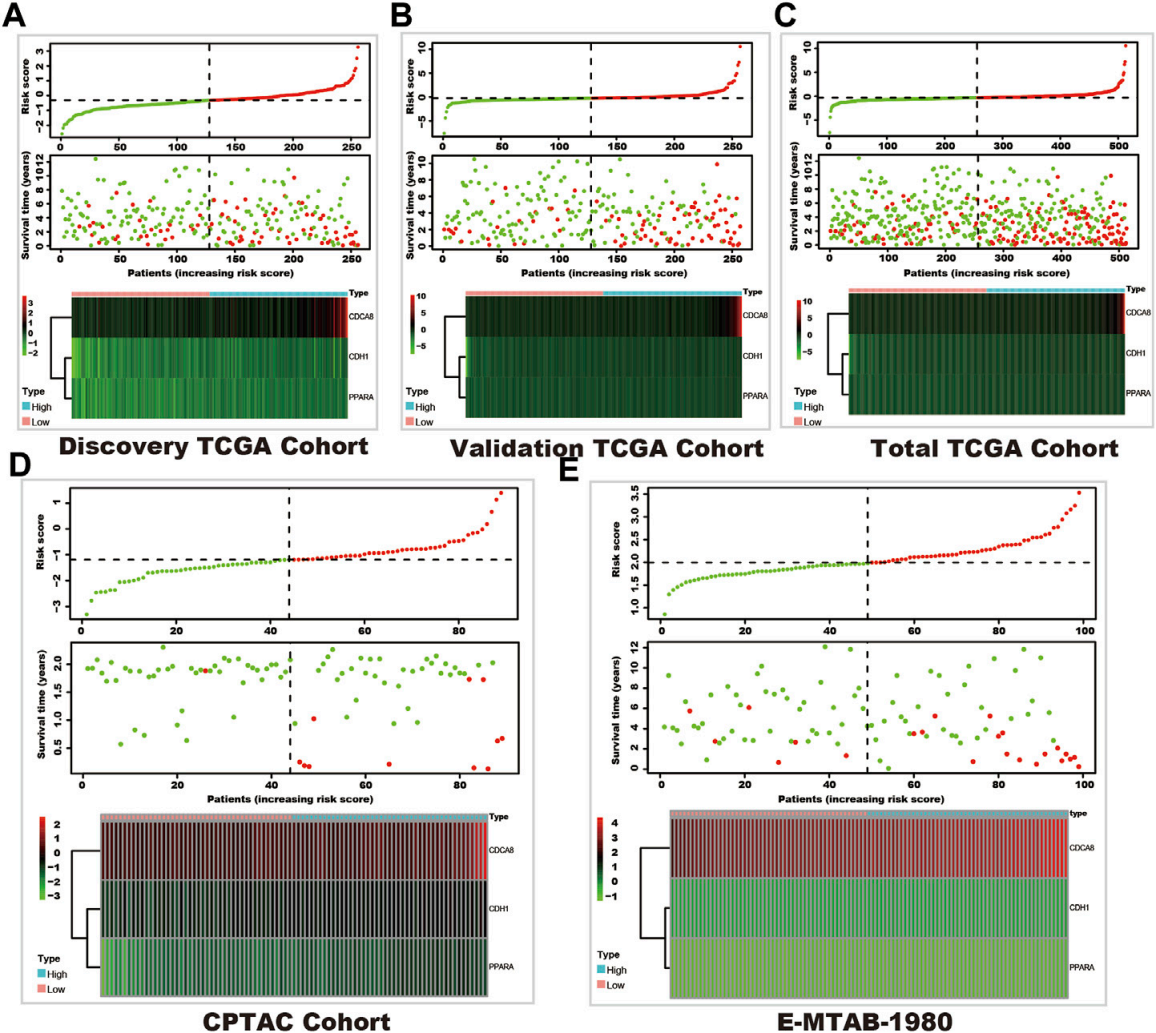
The survival and SUMOylation risk score distribution and SUMOylation risk model gene expression status. In each sub-figure, the upper panel shows the risk score distribution, the dashed horizontal line shows the median value of the risk score, and the dashed vertical line shows the patients with the median value of the risk score (green dots show the low-risk group, and red dots shows the high-risk group). The middle panel shows the survival status (red dots represent dead status, and green dots represent live status), and the bottom panel shows the heatmap of the SUMOylation risk model gene expression in each cohort. **(A)** Discovery TCGA cohort. **(B)** Validation TCGA cohort. **(C)** Total TCGA cohort. **(D)** CPTAC cohort. **(E)** E-MTAB-1980 cohort.

**FIGURE 2:**
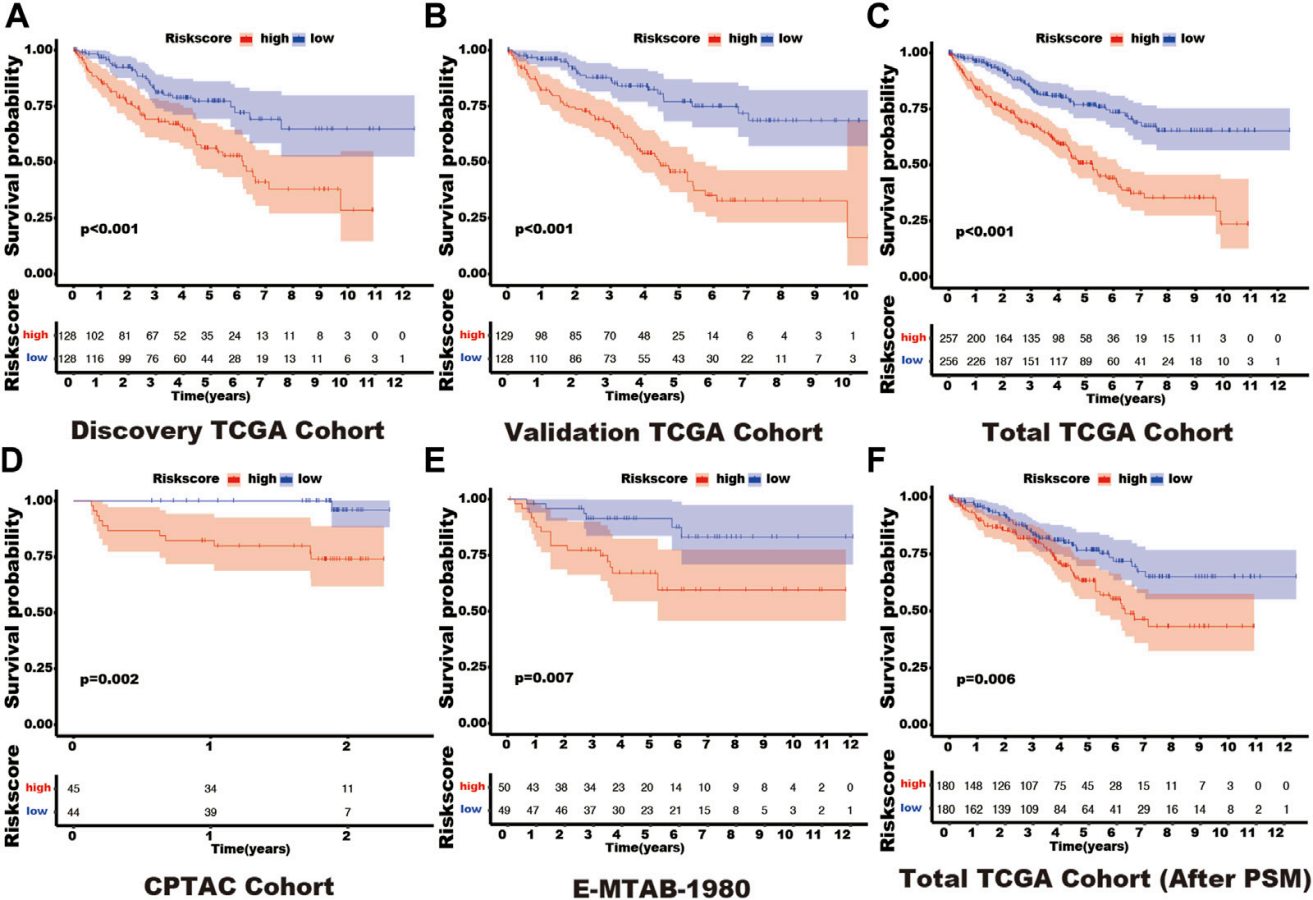
The different outcomes in various SUMOylation risk groups. In each figure, the red line represents the outcome of patients in the high SUMOylation risk group, the blue line shows the outcome of patients in the low SUMOylation risk group, and the box shows the live patient count during the entire follow-up period. Log-rank analysis was used to compare the outcomes of patients in different risk groups and presented by the Kaplan-Meier curve. **(A)** Discovery TCGA cohort. **(B)** Validation TCGA cohort. **(C)** Total TCGA cohort. **(D)** CPTAC cohort. **(E)** E-MTAB-1980 cohort. **(F)** Total TCGA cohort (After PSM). Propensity-matched analysis: PSM.

In these 29 genes, seven (AURKB, BIRC5, CDCA8, CDH1, NR1H3, PPARA, and TOP2A) were correlated with the prognosis of kidney cancer through univariate cox regression analysis using the discovery TCGA cohort, and five genes (AURKB, CDCA8, CDH1, NR1H3, and PPARA) were selected from Lasso regression (Supplementary Figure S2). In addition, three genes (CDCA8, CDH1, and PPARA) presented as independent factors of kidney cancer by multivariate cox regression (Supplementary Material). Finally, a SUMOylation risk model was constructed with the following equation: risk score = 0.413023054*CDCA8- 0.024059083*CDH1-0.12352751*PPARA. The patients’ risk scores in these five cohorts were computed, and patients in each cohort were divided into two risk groups. In the discover TCGA cohort, patients with high-risk scores often had a short survival time (Figure 1A), and log-rank analysis also showed that patients in the high-risk group had a significantly worse outcome (*p* < 0.001, Figure 2A). These findings were verified by the four other cohorts (validation TCGA cohort, total TCGA cohort, CPTAC cohort, and E-MTAB-1980 cohort) (Figures 1B–E; Figures 2B–E).

**FIGURE 3:**
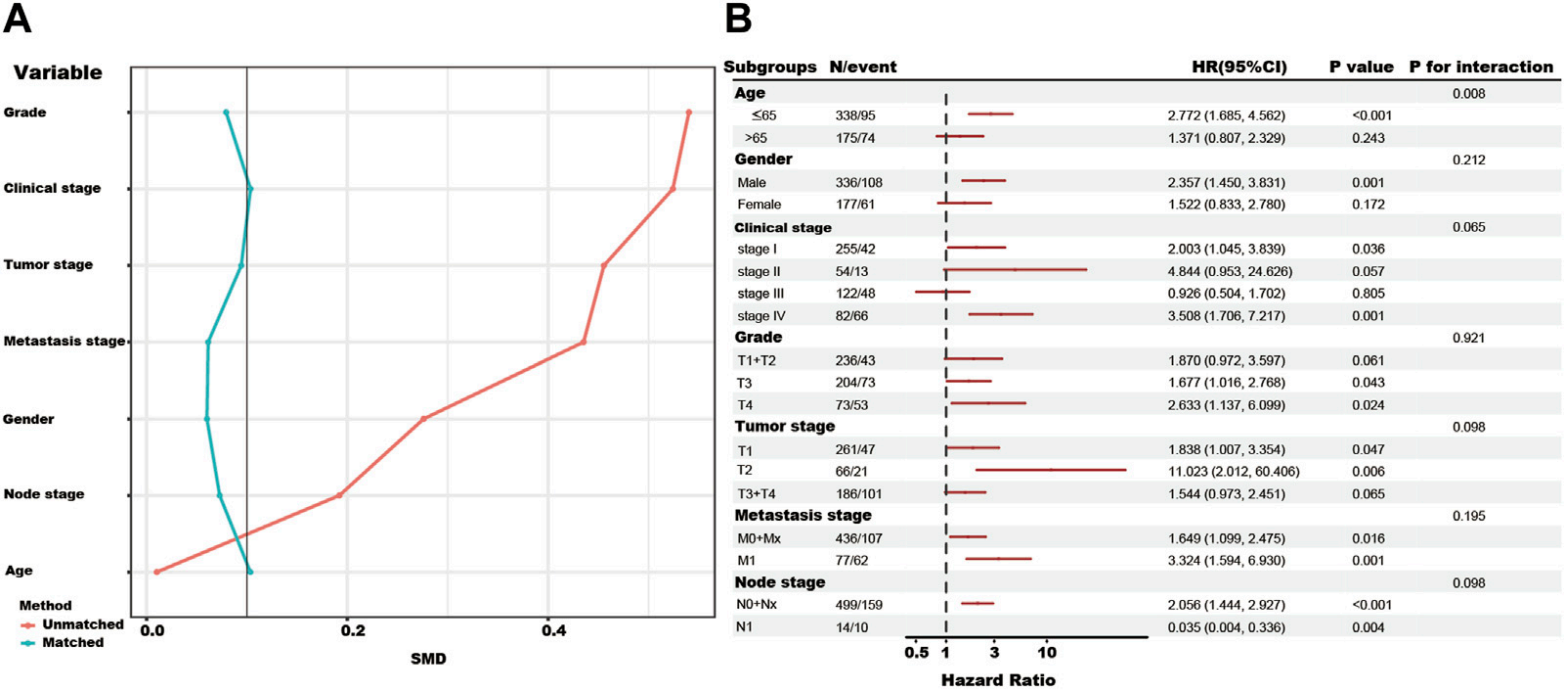
The propensity-matched analysis (PSM), subgroup analysis, and interaction test of SUMOylation risk group in the total TCGA cohort. **(A)** The red line showed the SMD value of the original total TCGA cohort. The green lines showed the SMD value of each variable after the adjustment of the PSM analysis. **(B)** The forest figure showed the result of the subgroup analysis and interaction test, and low-risk group as the reference.

In the total TCGA cohort, the high SUMOylation risk group is significantly correlated with Male patients (*p* = 0.003), high tumor stage (*p* < 0.001), metastasis stage (*p* < 0.001), clinical stage (*p* < 0.001), and tumor grade (*p* < 0.001) (Table 1). After the PSM analysis, each group has been assigned 180 patients, and there is no significant difference in clinical features between the high and low-risk groups (Table 1; Figure 3A). And the high-risk group still presents a poorer prognosis than the low-risk group (Figure 2F).

**TABLE 1:** The clinical feature in different risk group in total TCGA and total TCGA cohort after PSM.

	Original total TCGA cohort			PSM total TCGA cohort		
	Low risk group	High risk group	*p*-value	Low risk group	High risk group	*p*-value
	N = 256	N = 257		N = 180	N = 180	
Age (years)	60.36 ± 11.71	60.49 ± 12.41	0.908	59.63 ± 11.53	60.87 ± 12.51	0.327
Gender			0.003			0.648
Male	151 (58.98%)	185 (71.98%)		122 (67.78%)	127 (70.56%)	
Female	105 (41.02%)	72 (28.02%)		58 (32.22%)	53 (29.44%)	
Tumor stage			<0.001			0.839
T1	156 (60.94%)	105 (40.86%)		103 (57.22%)	101 (56.11%)	
T2	29 (11.33%)	37 (14.40%)		20 (11.11%)	24 (13.33%)	
T3	70 (27.34%)	105 (40.86%)		56 (31.11%)	53 (29.44%)	
T4	1 (0.39%)	10 (3.89%)		1 (0.56%)	2 (1.11%)	
Node stage			0.096			0.812
N1	118 (46.09%)	116 (45.14%)		85 (47.22%)	81 (45.00%)	
N2	3 (1.17%)	11 (4.28%)		1 (0.56%)	2 (1.11%)	
Nx	135 (52.73%)	130 (50.58%)		94 (52.22%)	97 (53.89%)	
Metastasis stage sstagestage s			<0.001			0.844
M1	222 (86.72%)	188 (73.15%)		156 (86.67%)	159 (88.33%)	
M2	19 (7.42%)	58 (22.57%)		16 (8.89%)	13 (7.22%)	
Mx	15 (5.86%)	11 (4.28%)		8 (4.44%)	8 (4.44%)	
Clinical stage			<0.001			0.810
Stage I	153 (59.77%)	102 (39.69%)		101 (56.11%)	99 (55.00%)	
Stage II	28 (10.94%)	26 (10.12%)		20 (11.11%)	24 (13.33%)	
Stage III	55 (21.48%)	67 (26.07%)		42 (23.33%)	44 (24.44%)	
Stage IV	20 (7.81%)	62 (24.12%)		17 (9.44%)	13 (7.22%)	
Grade			<0.001			0.900
Grade 1	9 (3.52%)	4 (1.56%)		3 (1.67%)	4 (2.22%)	
Grade 2	132 (51.56%)	91 (35.41%)		83 (46.11%)	82 (45.56%)	
Grade 3	100 (39.06%)	104 (40.47%)		80 (44.44%)	83 (46.11%)	
Grade 4	15 (5.86%)	58 (22.57%)		14 (7.78%)	11 (6.11%)	

PSM, propensity score matching analysis.

In addition, the subgroup and interaction analysis also show high-risk group associated with poor outcomes in the patients with the following features, ≤65 years, male, clinical stage I, clinical stage IV, grade III, grade IV, T1, T2, M0+Mx, M1 and N0+Nx. The contrary result in the N1 subgroup might be due to the limited number of patients (Figure 3B).

**FIGURE 4:**
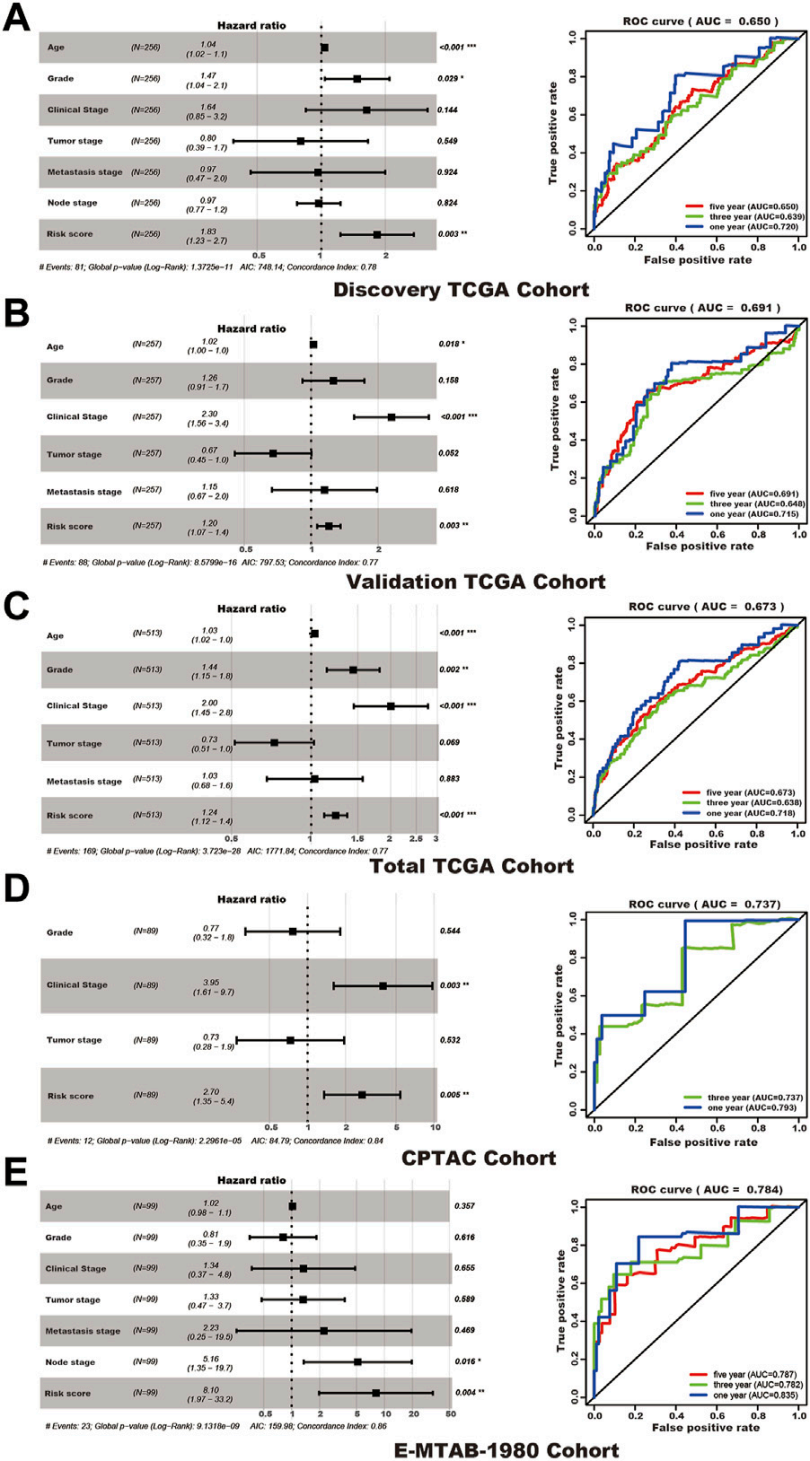
The SUMOylation risk score is an independent poor prognosis factor of kidney cancer patients. In each subfigure, the left panel shows the multivariate cox regression results, and the right panel presents the receiver operating characteristic curve of 1, 3, and 5 years (the 1 and 3 years of the CPTAC cohort). **(A)** Discovery TCGA cohort. **(B)** Validation TCGA cohort. **(C)** Total TCGA cohort. **(D)** CPTAC cohort. **(E)** E-MTAB-1980 cohort.

Although clinical features present in each cohort may vary in functions for predicting the survival status, the SUMOylation risk score could be an independent prognosis factor in all five cohorts and presented a good prognostic effect (Table 2; Figure 4).

**TABLE 2:** Univariate and Multivariate Cox regression reveals riskscore is an independent risk factor in kidney cancer.

Cohorts	Variables	Univariate cox regression		Variables	Multivariate cox regression	
		HR (95% CI)	*p*-value		HR (95% CI)	*p*-value
Discovery TCGA cohort	Age	1.035 (1.015–1.055)	**<0.001**	Age	1.038 (1.016–1.06)	**0.001**
	Gender	1.154 (0.732–1.817)	0.538			
	Grade	2.331 (1.715–3.168)	**<0.001**	Grade	1.475 (1.04–2.091)	**0.029**
	Clinical stage	1.706 (1.417–2.053)	**<0.001**	Clinical stage	1.635 (0.846–3.161)	0.144
	Tumor Stage	1.931 (1.508–2.472)	**<0.001**	Tumor Stage	0.801 (0.387–1.658)	0.549
	Metastasis stage	1.771 (1.248–2.515)	**0.001**	Metastasis stage	0.965 (0.466–1.998)	0.924
	Node stage	0.797 (0.638–0.995)	**0.045**	Node stage	0.974 (0.77–1.231)	0.824
	SRGs riskscore	2.718 (1.887–3.916)	**<0.001**	SRGs riskscore	1.83 (1.231–2.721)	**0.003**
Validation TCGA cohort	Age	1.024 (1.005–1.042)	**0.012**	Age	1.024 (1.004–1.045)	**0.018**
	Gender	0.951 (0.615–1.47)	0.82			
	Grade	2.207 (1.669–2.919)	**<0.001**	Grade	1.26 (0.914–1.735)	0.158
	Clinical stage	2.103 (1.733–2.552)	**<0.001**	Clinical stage	2.297 (1.561–3.379)	**<0.001**
	Tumor Stage	1.899 (1.521–2.37)	**<0.001**	Tumor Stage	0.675 (0.454–1.003)	0.052
	Metastasis stage	2.625 (1.916–3.596)	**<0.001**	Metastasis stage	1.148 (0.667–1.976)	0.618
	Node stage	1.011 (0.818–1.249)	0.92			
	SRGs riskscore	1.285 (1.171–1.41)	**<0.001**	SRGs riskscore	1.202 (1.065–1.356)	**0.003**
Total TCGA cohort	Age	1.029 (1.016–1.043)	**<0.001**	Age	1.032 (1.018–1.047)	**<0.001**
	Gender	1.049 (0.767–1.437)	0.764			
	Grade	2.284 (1.861–2.804)	**<0.001**	Grade	1.443 (1.146–1.816)	**0.002**
	Clinical stage	1.895 (1.659–2.166)	**<0.001**	Clinical stage	2.003 (1.45–2.769)	**<0.001**
	Tumor Stage	1.919 (1.626–2.264)	**<0.001**	Tumor Stage	0.726 (0.514–1.025)	0.069
	Metastasis stage	2.179 (1.728–2.748)	**<0.001**	Metastasis stage	1.032 (0.68–1.566)	0.883
	Node stage	0.905 (0.777–1.054)	0.2			
	SRGs riskscore	1.334 (1.236–1.439)	**<0.001**	SRGs riskscore	1.238 (1.12–1.368)	**<0.001**
CPTAC cohort	Age	1.014 (0.964–1.065)	0.596			
	Gender	1.318 (0.356–4.873)	0.679			
	Grade	2.603 (1.225–5.532)	**0.013**	Grade	0.765 (0.323–1.814)	0.544
	Clinical stage	3.853 (1.819–8.164)	**<0.001**	Clinical stage	3.95 (1.607–9.708)	**0.003**
	Tumor Stage	2.894 (1.287–6.506)	**0.01**	Tumor Stage	0.732 (0.275–1.949)	0.532
	Metastasis stage	0.781 (0.429–1.423)	0.42	Metastasis stage		
	Node stage	0.331 (0.1–1.102)	0.072	Node stage		
	SRGs riskscore	3.33 (1.867–5.939)	**<0.001**	SRGs riskscore	2.699 (1.349–5.402)	**0.005**
E-MTAB cohort	Age	1.044 (1.002–1.087)	**0.04**	Age	1.02 (0.978–1.063)	0.357
	Gender	0.441 (0.131–1.486)	0.187			
	Grade	2.982 (1.671–5.32)	**<0.001**	Grade	0.809 (0.353–1.852)	0.616
	Clinical stage	2.289 (1.654–3.167)	**<0.001**	Clinical stage	1.339 (0.371–4.829)	0.655
	Tumor Stage	2.564 (1.675–3.925)	**<0.001**	Tumor Stage	1.329 (0.475–3.72)	0.589
	Metastasis stage	6.113 (2.571–14.537)	**<0.001**	Metastasis stage	2.228 (0.255–19.504)	0.469
	Node stage	18.529 (7.024–48.876)	**<0.001**	Node stage	5.155 (1.351–19.68)	**0.016**
	SRGs riskscore	15.613 (5.516–44.194)	**<0.001**	SRGs riskscore	8.097 (1.973–33.22)	**0.004**

The bold values implied the correspondence variable was shown significant difference in Univariates or Multivariate Cox regression analysis.

**FIGURE 5:**
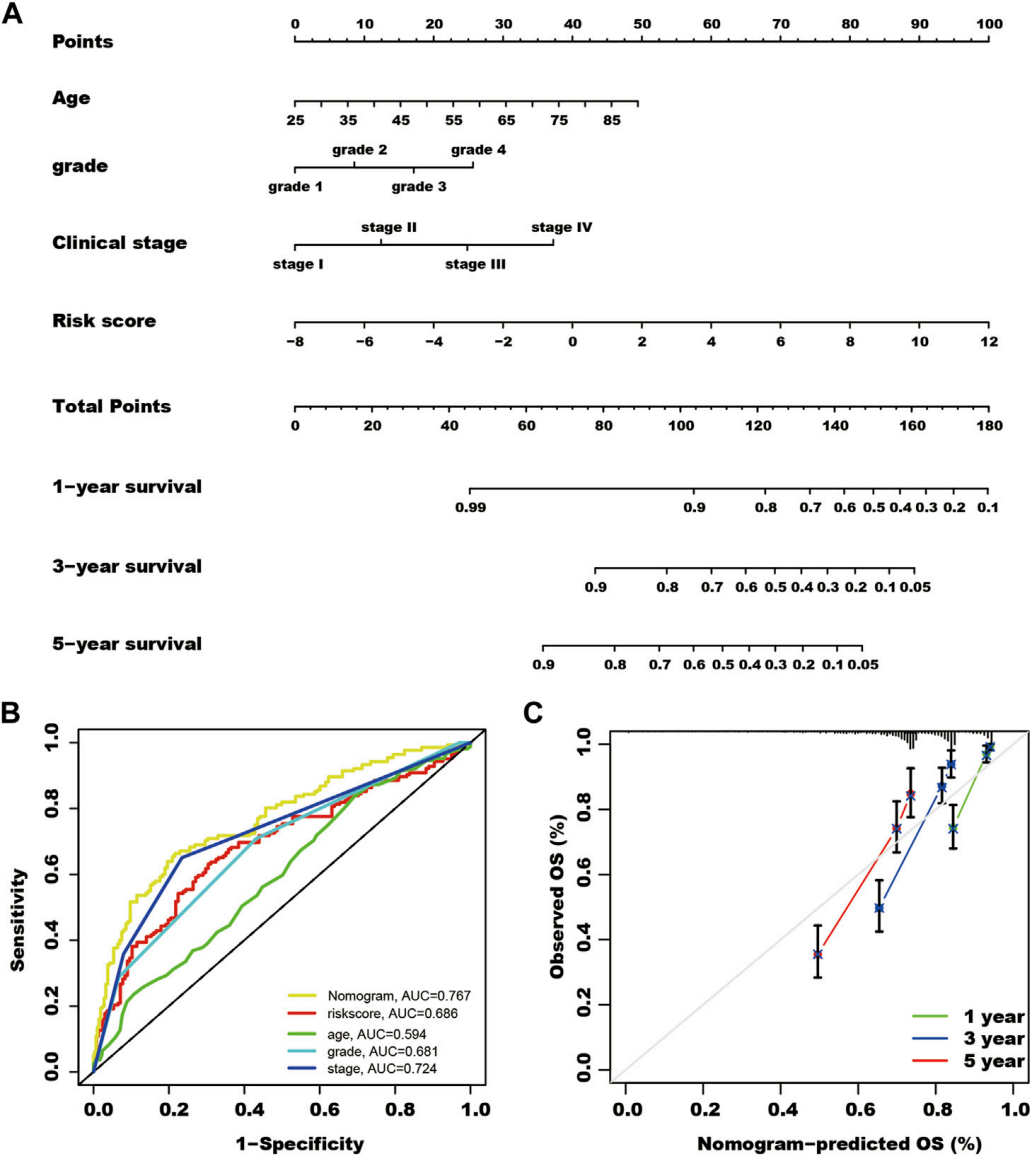
A nomogram for predicting the survival rate of kidney cancer patients. **(A)** Using multivariate cox regression results of the total The Cancer Genome Atlas cohort, patient age, Grade, clinical stage, and SUMOylation risk score show independent risk factors of patient outcomes. Each feature was assigned a value, and the total value of patients corresponded to values of 1-, 3- and 5-year survival rates. **(B)** The nomogram shows the highest AUC value of in multi-ROC analysis. **(C)** Calibration curves of the nomogram for predicting the outcome of 1, 3, and 5 years in the total TCGA cohort.

In the total TCGA cohort, age, clinical stage, Grade, and SUMOylation risk score presented as independent risk factors, and these four variables were used to build a nomogram to predict the survival rate of patients with kidney cancer (Figure 5).

### Different SUMOylation subgroups present various molecular features

**FIGURE 6:**
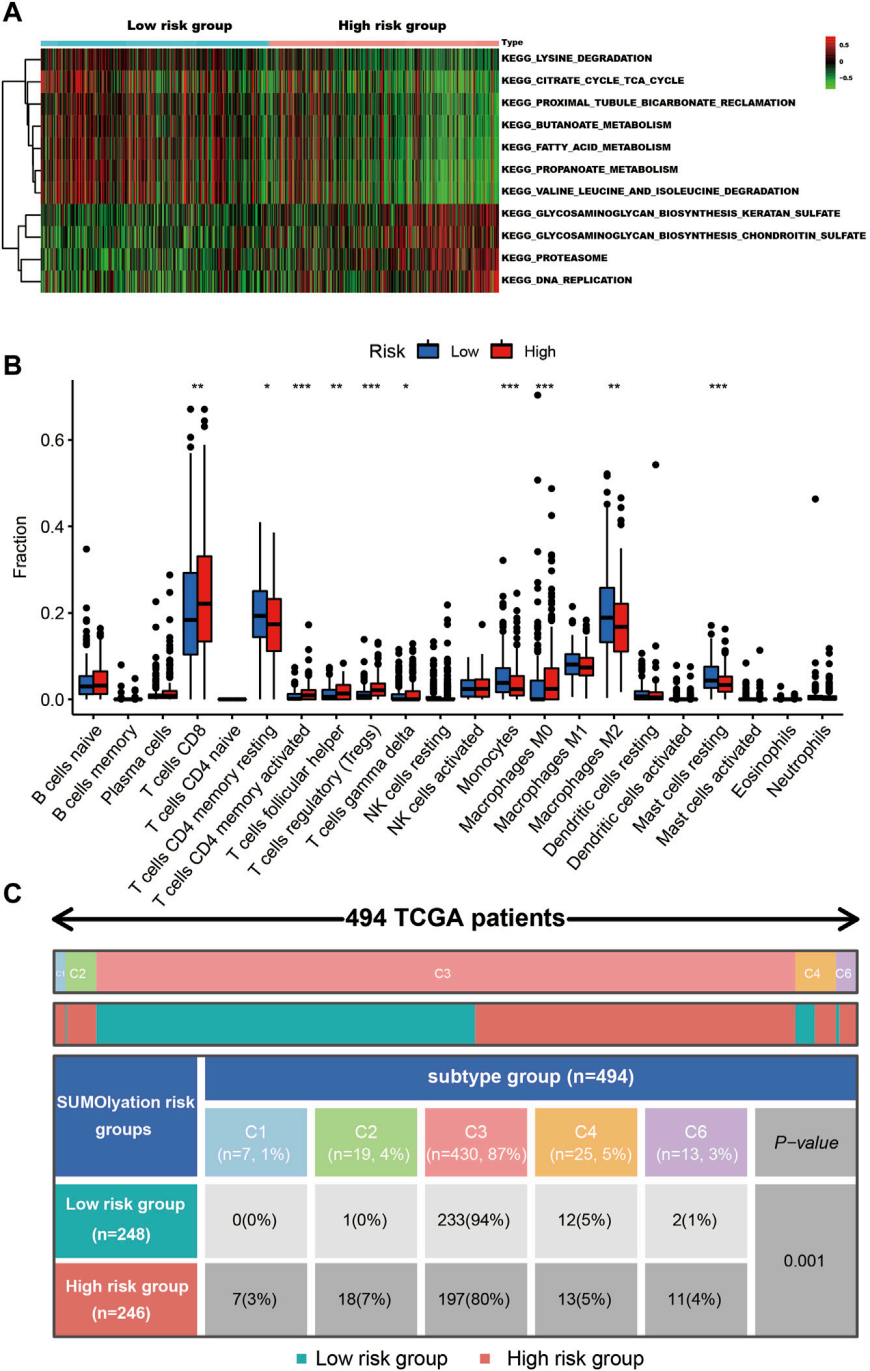
The cell signaling and immune status in the different risk groups of kidney cancer tissues. **(A)** The GSVA analysis shows seven pathways were up-regulated in the low-risk group, while four pathways were up-regulated in the high-risk group. **(B)** The different types of immune cells infiltrated in the tumor tissues, *** indicates *p* < 0.001, ** indicates *p* < 0.01, and * indicates *p* < 0.05. **(C)** The patients’ immune subtypes in the different risk groups. C1 (wound healing), C2 (IFN-g dominant), C3 (inflammatory), C4 (lymphocyte depleted), and C6 (TGF-b dominant).

GSVA analysis revealed the proteasome, DNA replication, glycosaminoglycan biosynthesis chondroitin sulfate, glycosaminoglycan biosynthesis keratan sulfate pathways were significantly highly enriched in kidney cancer tissues with the high-risk group (Figure 6A).

In the two groups (the high-risk and low-risk groups), 10 types of immune cell infiltration statuses were different. Among them, T cell CD8, T cells CD4 memory activated, T cells follicular helper, T cells regulatory (Tregs), T cells gamma delta, and macrophages M0, were higher in the tissues of the high-risk group, while T cells CD4 memory resting, monocytes, macrophages M2, and mast cells resting were higher in the low-risk group (Figure 6B). Tissues in the high-risk group presented higher ratios of C1, C2, C4, and C6 (total 20%) than tissues in the low-risk group (total 6%). Additionally, they had a lower C3 ratio (80%) than tissues in the low-risk group (94%) (Figure 6C).

### SUMOylation subgroups may be a biomarker of kidney cancer treatment

**FIGURE 7:**
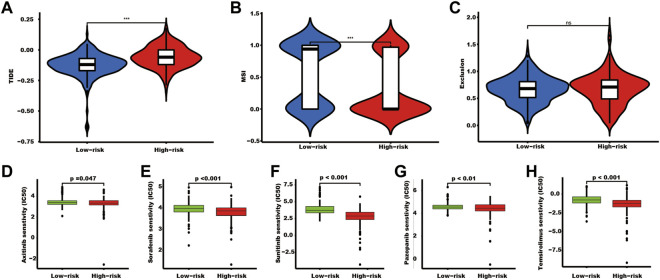
The TIDE score and targeted drug treatment sensitivity prediction in different risk groups. **(A–C)** TIDE, MSI, and exclusion scores of the two SUMOylation risk groups (*** indicates *p* < 0.001, ns indicates no significant difference). **(D–H)** Five targeted drugs half maximal inhibitory concentration prediction.

Tissues in the high-risk group showed a high TIDE score and a lower MSI score (Figure 7A). In addition, tumor tissues in the high-risk group had a lower IC50 of axitinib (*p* = 0.047), sorafenib (*p* < 0.001), sunitinib (*p* < 0.001), pazopanib (*p* < 0.01), and temsirolimus (*p* < 0.001) (Figure 7B).

### RNA and protein expression status of the SUMOylation risk model genes

**FIGURE 8:**
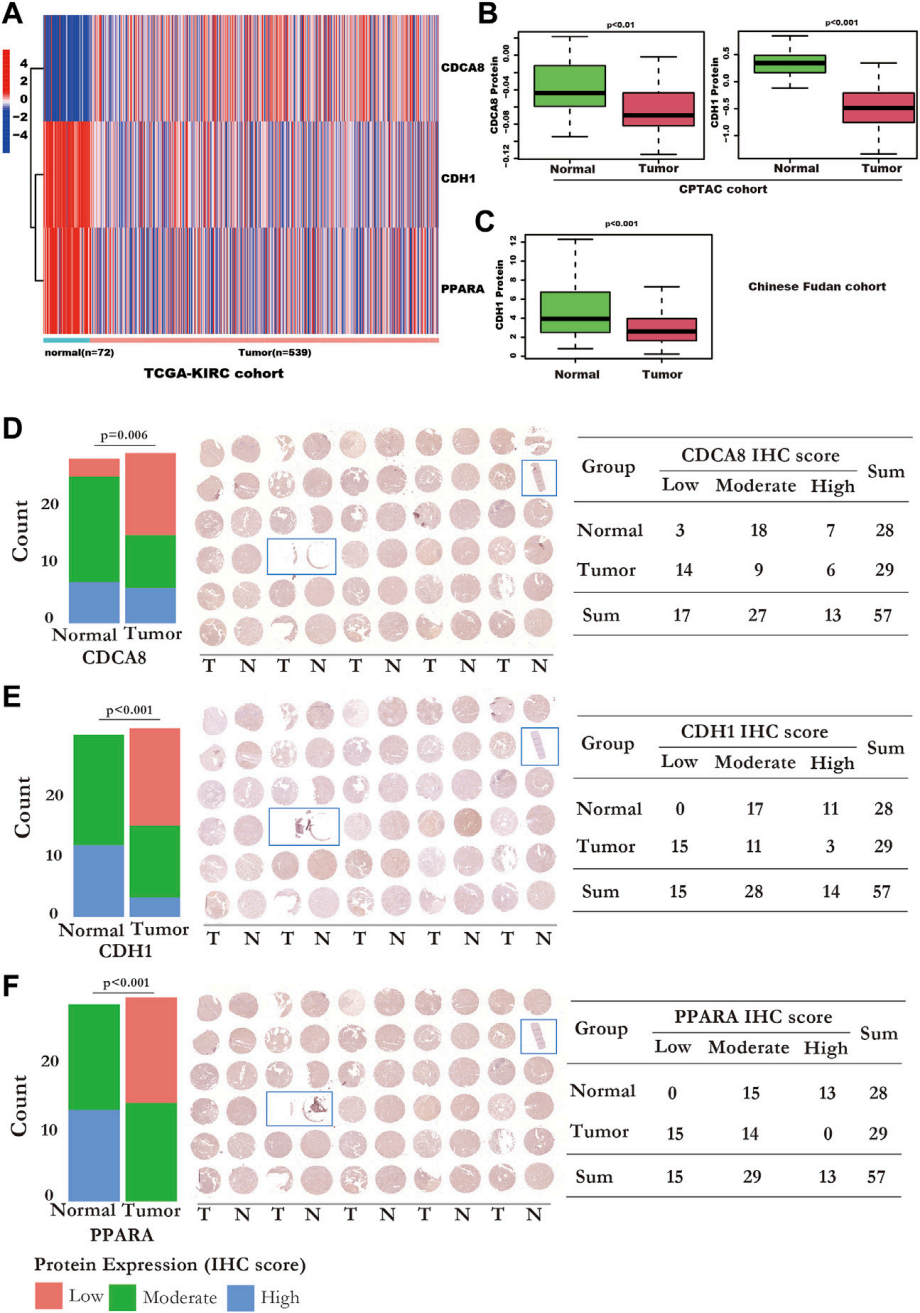
The RNA and protein expression of the SUMOylation risk model genes. **(A)** The RNA expression values of cell division cycle-related protein 8 (CDCA8) (*p* < 0.001), CDH1 (*p* < 0.001), and peroxisome proliferator-activated receptor alpha (PPARA) genes (*p* < 0.001), between normal and kidney cancer tissues from the TCGA-KIRC dataset. **(B)** CDCA8 and CDH1 protein expression of the normal and tumor tissues in the National Cancer Institute’s Clinical Proteomic Tumor Analysis Consortium cohort dataset. **(C)** CDH1 protein expression status in Chinese Fudan cohort. **(D)** The IHC detection of CDCA8 protein expression in TMA tissues. Left panel: the bar plot shows the CDCA8 expression status detected by IHC. Middle panel: The whole TMA slide of CDCA8 detection; the tissue with the blue rectangle means these tissues were excluded from statistics because the tissue was too little. Right panel: displayed detailed CDCA8 statistic data. **(E)** The IHC detection of CDH1 protein expression in TMA tissues. Left panel: the bar plot shows the CDH1 expression status detected by IHC. Middle panel: The whole TMA slide of CDH1 detection; the tissue with the blue rectangle means these tissues were excluded from statistics because the tissue was too little. Right panel: displayed detailed CDH1 statistic data. **(F)** The IHC detection of PPARA protein expression in TMA tissues. Left panel: the bar plot shows the PPARA expression status detected by IHC. Middle panel: The whole TMA slide of PPARA detection; the tissue with the blue rectangle means these tissues were excluded from statistics because the tissue was too little. Right panel: displayed detailed PPARA statistic data.

At the RNA level, CDCA8 was overexpressed, while CDH1 and PPARA were downregulated in kidney cancer tissues, in TCGA-KIRC (Figure 8A). PPARA was downregulated at both the RNA (Figure 8A) and protein levels (Figure 8F). CDH1 was downregulated in cancer tissues at both the RNA (Figure 8A) and protein levels in the CPTAC (Figure 8B) and Chinese Fudan cohort which detecting by proteome sequencing (Figure 8C), and TMA tissues (Figure 8E). However, CDCA8 protein was downregulated in the CPTAC kidney cancer tissues (Figure 8B) and TMA tissues (Figure 8D), which is contrary to the RNA level (Figure 8A).

## Discussion

Here, we analyzed the SUMOylation-related gene expression status at the RNA level in kidney cancer tissues and built a risk model using three SUMOylation genes, CDH1, CDCA8, and PPARA, to indicate the outcomes based on differentially expressed SUMOylation genes. The risk model also presented as a potential biomarker to reflect the sensitivity of targeted therapy and immune status in tumor tissues. Our study analyzed the protein expression data of these three SUMOylation risk model genes.

Few studies previously focused on the role of SUMOylation in kidney cancer. Overexpression of SUMO-Specific Protease 1 (SENP1), a SUMO protease, could reduce the SUMOylation and ubiquitination of HIF2α, and then increase the local invasion and metastasis capacity of ccRCC cells (Lee et al., 2022). While SENP1 deficiency could lead to deSUMOyation of HIF1α and then inhibit cell proliferation (Dong et al., 2016). In addition, the SUMOylation of hypoxia-associated factor (HAF) could mediate HIF1α degradation, and promoter HIF2α-dependent transcription, and then promote ccRCC development and morbidity (Koh et al., 2015). In our study, the GSVA analysis showed proteasome pathways were significantly highly enriched in kidney cancer tissues in the high-risk group (Figure 6A). Proteasomes take part in the degrading protein process, and it was involved in lots of cellular functions, the dysregulation of proteasomes could result in uncontrolled growth, immune escape, drug resistance, and EMT of cancer (Chen et al., 2017). The poor prognosis of kidney patients in the high-risk group might be associated with the abnormal proteasome in tumor tissue.

CDH1 is also known as E-Cadherin, and its mutation has proven to correlate with tumorigenesis, progression, and invasion of gastric, breast, and colorectal cancers. In addition, the CDH1 protein is a subunit of an E3 ubiquitin ligase, an anaphase-promoting complex or cyclostome (APC/C), and can participate in the mechanism of P53 regulating the G2 checkpoint through SUMOylation (Wang et al., 2020). Previous studies have shown that HIF can mediate suppression of CDH1 gene transcription, and result in the downregulation of E-cadherin in VHL-deficient kidney tumor tissues. In addition, one study showed that hypermethylation of the E-cadherin promoter also contributes to the downregulation of CDH1 (Dulaimi et al., 2004; Esteban et al., 2006). These results may partly explain the reason that CDH1 was downregulated both at the RNA and protein levels. Moreover, Zhang et al. (2017) revealed that reduced E-cadherin in kidney cancer could facilitate tumor progression by activating the WNT/β-catenin pathway. PPARA is a transcription factor, and it can modulate lipid, glucose, and amino acid metabolism, regulate inflammation, and display a tumor suppressor or oncogene role in many cancer types (Tan et al., 2021). PPARA can be SUMOylated and results in the function nuance regulation (Wadosky and Willis, 2012). Indeed, the SUMOylation of PPARA should result in a decrease in its transcriptional activity. In kidney cancer, inhibiting PPARA through an antagonist or siRNA could induce apoptosis and cell cycle arrest of kidney cancer cell lines, and it has been proven to be a diagnostic and prognostic marker for ccRCC (Abu Aboud et al., 2013). Although our study showed that PPARA is downregulated in kidney cancer tissues, Omran et al. revealed that PPARA protein expression was correlated with Grade, where it had a higher expression in grade 4 tissues than in grade 1 tissues (Abu Aboud et al., 2013). The CDCA8 gene encodes a protein that takes part in the composing of chromosomal passenger complex. CDCA8 is upregulated in bladder and prostate cancer tissues, and knockdown of CDCA8 inhibits tumor cell proliferation (Gao et al., 2020; Xu et al., 2022). In addition, CDCA8 expression status could predict the prognosis of prostate and liver cancers (Shuai et al., 2021; Xu et al., 2022). CDCA8 was identified as a core gene involved in the metastasis of ccRCC cells. (Peng et al., 2020). The function of CDCA8 in kidney cancers should be further investigated.

In this study, we determined that CDH1 (Figures 8A–C, E), and PPARA (Figures 8A, F) were downregulated both at the RNA and protein levels. However, CDCA8 protein was downregulated in CPTAC kidney cancer tissues assessed by proteome sequencing (Figure 8B) and in kidney cancer TMA tissues by the IHC method (Figure 8D), which is contrary to the RNA level (Figure 8A). Many reasons might contribute to the contradiction between the protein and RNA expression levels of CDCA8. Studies had revealed that MiR-133a-3p played crucial roles in the regulation of CDCA8 in oesophageal cancer (Wang et al., 2022), and miR-133b regulated the CDCA8 expression in lung adenocarcinoma (Hu et al., 2021) through a post-transcriptional regulation manner. However, the mechanism of the contrary expression level of RNA and protein in kidney cancer has not been well discussed, which should be studied in the future.

Our SUMOylation risk model indicated that patients in the high-risk group have a worse outcome. The immune subtype analysis revealed a significantly higher ratio of C1, C2, C4, and C6 subtypes and a lower C3 subtype in the high-risk model group. The previous study proposed that patients in the C3 subtype often had the best prognosis of all subtypes, while patients in the C6 subtype had the worst outcome. Different outcomes of the distinct SUMOylation risk group might be attributable to the varying immune statuses in tumor tissues, which were assessed by the immune subtype and immune cell analysis.

A high TIDE score was associated with the immune escape capacity of tumor cells, while a low IC50 might indicate a sensitivity to drug treatment. In this study, tissues of the high-risk group presented a high TIDE score and a low IC50 of the five targeted drugs, which implied that patients with high-risk scores might be more suitable for receiving the ICB plus targeted agent treatment as the first-line treatment.

In our study, we examined the RNA expression status of SUMOylation genes in kidney cancer tissues and constructed and verified a SUMOylation prognosis model for predicting the outcome of kidney cancer using three databases and five cohorts. In conclusion, the SUMOylation risk model may be a biomarker for the selection of treatment drugs for kidney cancer.

## Data availability statement

The datasets presented in this study can be found in online repositories. The names of the repository/repositories and accession number(s) can be found in the article/Supplementary Material.
