# Supplementary material for: A novel risk model of three SUMOylation genes based on RNA expression for potential prognosis and treatment sensitivity prediction in kidney cancer
Source: Front Pharmacol. 2023 May 2;14:1038457. doi: 10.3389/fphar.2023.1038457 (PMC10185777; doi:10.3389/fphar.2023.1038457)
Supplement: Supplementary file 2 [file DataSheet1.docx]

**A novel three SUMOylation genes risk model with the function of potential prognosis and treatment sensitivity prediction for Kidney Cancer**

**Authors:**

Song-chao Li^1^, Li-jie Yan^2^, Xu-liang Wei^2^, Zhan-kui Jia^1^, Jin-jian Yang^1^, Xiang-hui Ning^1*^

**Affiliation:**

^1^ Department of Urology, the First Affiliated Hospital of Zhengzhou University, Zhengzhou, China.

^2^ Institute of Pharmaceutical Science, Zhengzhou University, Zhengzhou, China.

***Correspondence:**

Xiang-hui Ning, M.D., Ph.D.

Department of Urology, The First Affiliated Hospital, Zhengzhou University,

No.1 Eastern Jianshe Road, Er Qi District,

Zhengzhou, Henan 450052, P.R China.

E-mail: ningxianghui@126.com

**Keywords:**

Kidney cancer, prognosis, Sumoylation, targeted therapy, immune therapy


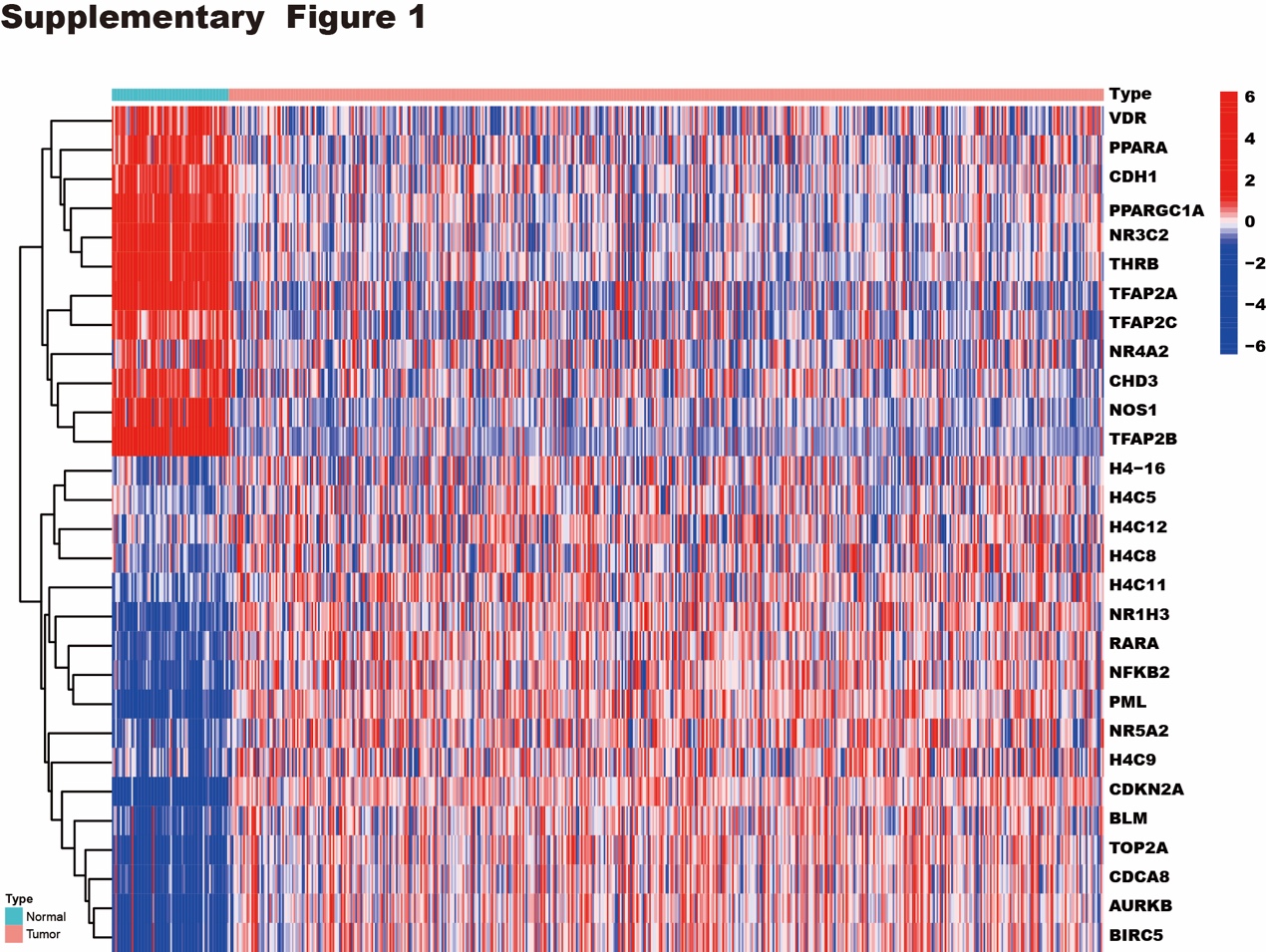


Supplementary Figure 1 legend: The differentially expressed SUMOlyation related genes in TCGA KIRC cohort..


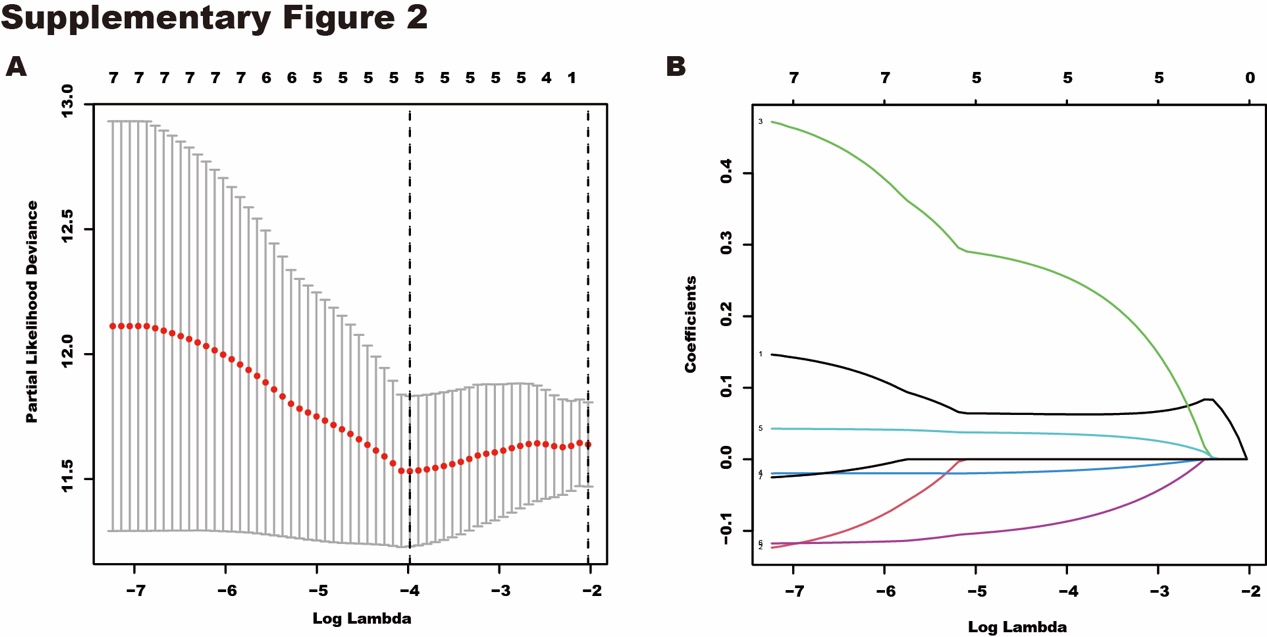


Supplementary Figure 2 legend: LASSO regression analysis of SUMOlyation risk model genes.
